# Modal Coupling Effect in a Novel Nonlinear Micromechanical Resonator

**DOI:** 10.3390/mi11050472

**Published:** 2020-04-29

**Authors:** Kuo Lu, Qingsong Li, Xin Zhou, Guoxiong Song, Kai Wu, Ming Zhuo, Xuezhong Wu, Dingbang Xiao

**Affiliations:** 1 College of Intelligence Science and Technology, National University of Defense Technology, Changsha 410073, China; lukuo13@nudt.edu.cn (K.L.); zhouxin11@nudt.edu.cn (X.Z.); songguoxiong14@nudt.edu.cn (G.S.); wukai@nudt.edu.cn (K.W.); zhuoming@nudt.edu.cn (M.Z.); xzwu@nudt.edu.cn (X.W.); 2 Laboratory of Science and Technology on Integrated Logistics Support, National University of Defense Technology, Changsha 410073, China

**Keywords:** nonlinear effect, mechanical nonlinearity, stiffness hardening, modal coupling, modal interaction, electrostatic coupling, MEMS resonator

## Abstract

Capacitive micromechanical resonators share electrodes with the same bias voltage, resulting in the occurrence of electrostatic coupling between intrinsic modes. Unlike the traditional mechanical coupling, the electrostatic coupling is determined by the structural electric potential energy, and generally, it only occurs when the coupling modes operate in nonlinear regions. However, previous electrostatic coupling studies mainly focus on the stiffness softening region, with little attention on the opposite stiffness hardening condition. This paper presents a study on the electrostatic modal coupling effect in the stiffness hardening region. A novel capacitive micromechanical resonator with different modal nonlinearities is designed and fabricated. It is demonstrated that activating a cavity mode can shift the fundamental resonance of the manipulated mode by nearly 90 times its mechanical bandwidth. Moreover, the frequency shifting direction is found to be related to the manipulated mode’s nonlinearity, while the frequency hopscotch is determined by the cavity mode’s nonlinearity. The electrostatic coupling has been proven to be an efficient and tunable dynamical coupling with great potential for tuning the frequency in a wide range. The modal coupling theory displayed in this paper is suitable for most capacitive resonators and can be used to improve the resonator’s performance.

## 1. Introduction

With the development of advanced lithography and micromachining processes, the sizes of micro-electro-mechanical-system (MEMS) resonators are constantly shrinking, expanding their applications in various fields such as RF filters, accelerometers, gyroscopes, pressure sensors, and so on [1,2,3,4,5,6,7,8]. Micro-nano resonators have become an important direction for the development of next-generation sensors by virtue of their small size, low cost, and superior performance, which has stimulated the research interest of many research groups [9]. However, structural nonlinearity is also introduced under the influence of the scale effect. The resonant structure is easy to work in a nonlinear state at the micro-nano scale, which greatly restricts the performance of resonators [10]. Due to the existence of the scale effect, MEMS resonators exhibit typical nonlinear characteristics [11,12,13], making their responses more complex.

According to different manifestations, the main nonlinearities in resonators are divided into two categories, the mechanical nonlinearity and the electrostatic nonlinearity [14,15,16,17]. The mechanical nonlinearity is mainly caused by the structure’s nonlinear elastic restoring force. When the mechanical nonlinearity occurs, the resonator’s frequency will increase with its response amplitude, showing a stiffness hardening characteristic. On the contrary, the electrostatic nonlinearity is common in capacitive resonators, especially when the gap between the resonant structure and electrodes changes significantly. The electrostatic nonlinearity is mainly induced by nonlinear electrostatic forces. It seems that the resonator’s stiffness becomes smaller so that it is used to describe the stiffness softening effect. It is precisely because of the existence of various nonlinear effects that the resonator’s response becomes complicated and exhibits rich physical effects. As a result, studying the nonlinear characteristics of the resonator has become an attractive research area.

The mutual coupling and interactions between two distinct modes are typical representatives of the frontier research in the resonator’s mechanism and applications, which has been studied for decades [18]. The appearance of the modal coupling effect means that there is an energy exchange between the resonator’s different modes, and the originally independent relationship between these modes is destroyed. Generally, the modal coupling effect inside a resonator is determined by both the resonant structure and its multi-physics fields. Modal coupling effects based on different mechanisms, such as physical mechanical linkages, dielectric coupling tension-induced parametric coupling, internal resonance, and electrostatic coupling, have been extensively studied [19,20,21,22,23]. As for the capacitive micromechanical resonator, due to the existence of shared structure and electrodes, the response of any mode will affect the electric potential energy of the entire resonator system, so that these different modes are related and coupled with each other.

In a capacitive micromechanical resonator, the electrostatic nonlinearity is common since its resonant structure is driven and detected by electrical methods. As a result, its modal coupling studies mainly focus on the condition where the coupled modes both have electrostatic nonlinearities [19]. There is little analysis on the situation where the coupling modes present different types of nonlinearities. In order to complete the theoretical system of modal coupling effect in capacitive resonators, the coupling between different nonlinear modes needs further research and analysis.

In this paper, the modal coupling effect in a nonlinear micromechanical resonator is theoretically analyzed and associated with experimental results. A novel vacuum-packaged capacitive micromechanical resonator is designed and fabricated, whose different modes exhibit different nonlinearities, to analyze the modal coupling effect between different nonlinear modes. This paper is organized as follows. The basic structure of the capacitive resonator and controlling circuits are briefly introduced in Section 2. At the same time, the finite element method (FEM) is used to simulate the resonator’s operating modes. Then, based on the multi-scale method [24], the modeling analysis of the resonator’s nonlinear characteristics and modal coupling effect is carried out in Section 3. Furthermore, Section 4 presents the experimental results of the resonator’s nonlinearities and frequency shifting effects. Finally, the basic principles for the nonlinear modal coupling effect in capacitive MEMS resonators are concluded in Section 5.

## 2. Materials and Methods

### 2.1. Structure Description

**Figure 1:**
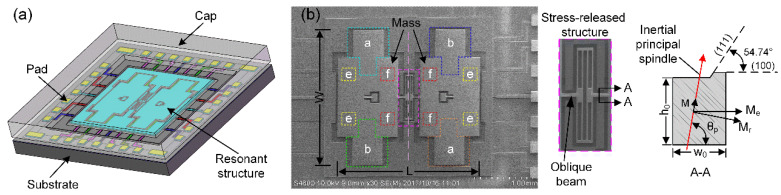
(**a**) The schematic of the vacuum-packaged capacitive micromechanical resonator, containing the resonant structure, the substrate, electrodes, and the cap; (**b**) The scanning electron micrograph of partial fabricated structure and electrodes.

A novel capacitive micromechanical resonator is specially designed and fabricated in this work. It is a tuning fork resonator consisting of two proof-masses, two anchors, one stress-released structure, and one oblique beam [25,26]. The resonant structure is bonded to the substrate through two anchors. There is an electrode gap between its sensitive structure and electrode layer after the bonding process. A variety of electrodes are arranged on the electrode layer for driving, sensing, and tuning its motion state. The main structural dimensions of this resonator are: thickness *T* is 40 μm, length *L* is 3 mm, width *W* is 3 mm, the spindle azimuth angle of the oblique beam *θ_p_* is 88.5°, and the capacitance gap *d*_0_ is 2 μm. In order to reduce the air damping, the vacuum-packaging process is applied, maintaining the resonator in a vacuum of 0.1 Pa. Figure 1 shows the schematic diagram of the resonator and its scanning electron micrograph.

**Figure 2:**
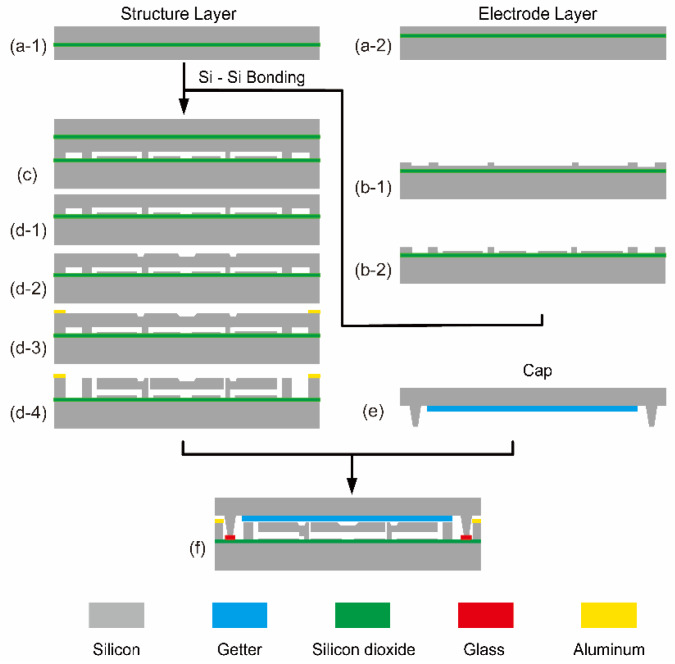
The fabrication process of the wafer-level vacuum packaged capacitive micromechanical resonator. (**a**) SOI wafers for the structure and the electrode layer fabrication; (**b**) Etch the electrode layer using deep reactive ion etching (DRIE); (**c**) Silicon-Silicon bond the structure’s SOI wafer and the electrode layer’s wafer together; (**d**) Thin the back surface of the structure SOI by mechanical chemical polishing (CMP), remove the oxide layer in solution, fabricate the oblique beam and sensitive structure using wet etching, and then aluminum pads are fabricated by physical vapor deposition (PVD), finally release the structure using DRIE; (**e**) The cap with the getter; (**f**) Wafer-level vacuum packaging process: using the glass paste to connect the resonant structure and the cap.

The primary process of the resonator includes dry etching, wet etching, silicon-silicon bonding, and wafer-level vacuum packaging. Firstly, the electrode layer was manufactured on a silicon-on-insulator (SOI) wafer. The desired electrode shape was obtained under a mask layer by using the dry etching. Afterward, wet etching (machining the oblique beam) and dry etching methods were applied to fabricate the resonator’s sensitive structure on another SOI wafer, which had been bonded to the former one. Finally, a cap prepared with a getter was bonded to the structural wafer, and after the laser dicing, a single vacuum packaged resonator could be obtained. The detailed process is illustrated in Figure 2.

### 2.2. Modal Simulation

**Figure 3:**
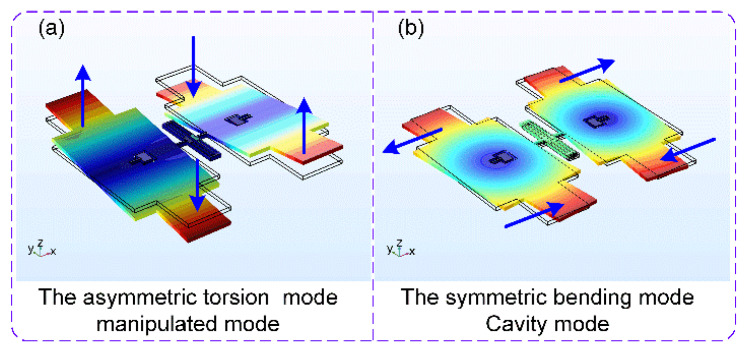
The modal simulation results of the capacitive micromechanical resonator. (**a**) The resonator’s asymmetric torsion mode; (**b**) The resonator’s symmetric bending mode.

The mode is an inherent vibrational characteristic of the structural system, and each mode has a specific natural frequency, damping ratio, and mode shape. As for the capacitive micromechanical resonator used in this work, its motion mainly includes the out-plane oscillation and the in-plane oscillation. This paper focuses on the resonator’s asymmetric torsion mode and symmetric bending mode, corresponding to its out-plane oscillation and in-plane oscillation, respectively. With the help of finite element simulation software, it is easy for us to get the information of the resonator’s different modes. The modal simulation results are displayed in Figure 3. It is obvious that these two modes have different kinds of motions, namely the torsional motion and the bending motion.

Using the sweeping circuits, we can get the actual fundamental frequencies of the resonator’s different modes, shown in Table 1. Compared with simulation results, there are some differences in the measured natural frequencies, which are mainly caused by machining errors and simulation accuracy errors. Within a certain range, these errors are unavoidable but acceptable. In order to better explore the nonlinear effect, in the following simulation and experimental processes, we use the natural frequency obtained by the experimental test as the reference value.

### 2.3. Controlling Circuits

**Figure 4:**
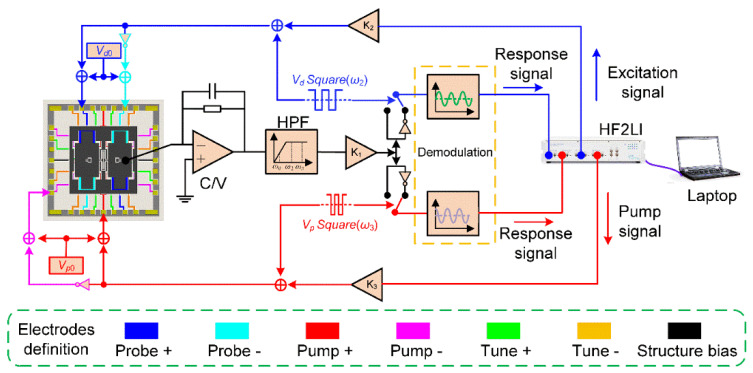
The schematic diagram of the controlling circuits.

The electrodes are connected to external pads through the lead wire layer. As a result, it is possible to flexibly select electrodes for different needs. To carry out the dynamic modal coupling experiments, a Zurich lock-in amplifier (HF2LI) is used to produce the excitation signal and pump signal at the same time. Then the resonator’s response signal enters the lock-in amplifier for analysis after demodulation. The schematic diagram of controlling circuits is displayed in Figure 4. The electrodes shown with the same color in the figure will be applied with the same electrical signal.

## 3. Nonlinear and Modal Coupling Theory

### 3.1. Nonlinear Effect

Previous researches demonstrated that the main nonlinearities in capacitive micromechanical resonators could be defined as the mechanical nonlinearity and the electrostatic nonlinearity [27]. The mechanical nonlinearity is mainly caused by the structure’s nonlinear elastic restoring force, while its electrostatic nonlinearity is the product of electrostatic forces.

Considering these nonlinearities, the simplified nonlinear dynamic model of the capacitive micromechanical resonator could be represented by the Duffing equation as shown below [28]:

(1) $$I\ddot{\phi }+c\dot{\phi }+(k_{0}+k_{1})\phi +k_{2}\phi^{2}+k_{3}\phi^{3}=M$$

where *I* is the inertia moment of the resonator, *c* is the damping coefficient, *M* is the excitation torque, *k*_0_ is the inherent elastic coefficient, *k*_1_, *k*_2_, and *k*_3_ are the first order, the second order, and the third order nonlinear elastic coefficient, respectively. These nonlinear elastic coefficients are determined by the combination of the resonator’s mechanical and electrostatic properties. As for the capacitive micromechanical resonator used in this work, its second order nonlinear elastic coefficient is small enough to be ignored, which would be analyzed in the following part.

Using the multi-scale method [24], we can get the system’s amplitude-frequency equation:

(2) $$a^{2}=\frac{M_{0}^{2}}{4I^{2}\omega_{0}^{2}[\frac{\omega_{0}^{2}}{4Q^{2}}+{\left(\Omega -\omega_{0}-\kappa a^{2}\right)}^{2}]}$$

Here,

(3) $$\kappa =\frac{3k_{3}}{8(k_{0}+k_{1})}\omega_{0}$$

**Figure 5:**
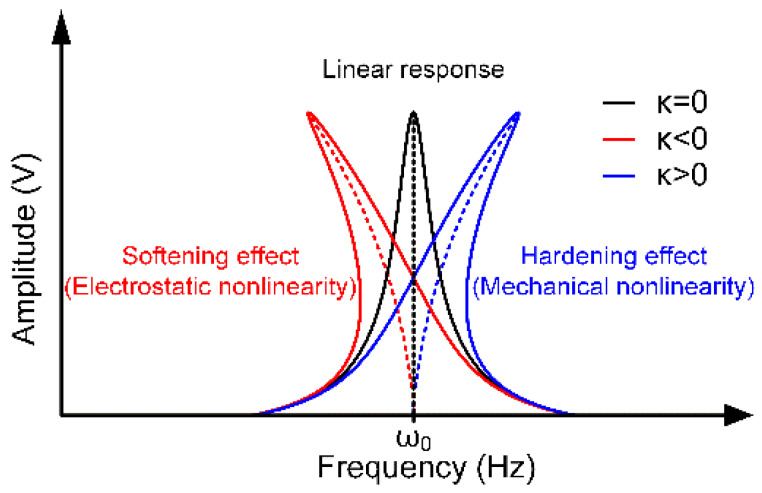
The resonant structure’s linear and nonlinear amplitude-frequency curves.

It is obvious that with the change of *κ*, the resonator’s response will exhibit different nonlinear properties as shown in Figure 5. In the linear regime (*κ* = 0), the response presents a typical Lorentzian shape. However, it is demonstrated that the relationship between the resonator’s amplitude and frequency is no longer a simple one-to-one correspondence in the nonlinear condition. When *κ* < 0, its response curve will deflect to the left, which is called the stiffness softening effect mainly caused by the electrostatic nonlinearity. By contrast, when *κ* > 0, this curve will deflect to the right, which is defined as the stiffness hardening effect induced by the mechanical nonlinearity. Actually, the electrostatic nonlinearity and mechanical nonlinearity coexist in a resonator, and the final form of the response depends on the dominant factor in these two nonlinearities.

The specially designed resonator’s motion mainly includes the out-plane and in-plane oscillation, whose representative is the asymmetric torsion mode and symmetric bending mode, respectively. Therefore, the nonlinear characteristics of these two modes are analyzed in the following parts.

#### 3.1.1. Mechanical Nonlinear Elastic Coefficients

**Figure 6:**
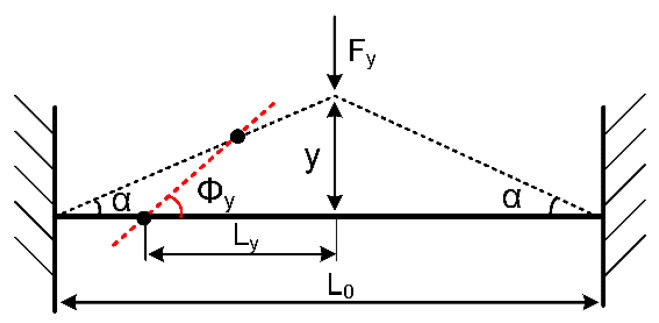
The clamped-clamped beam vibration model.

The mechanical nonlinearity is mainly caused by the structure’s nonlinear elastic restoring force. This type of nonlinearity is derived from the geometric structure’s nonlinear deformation at large displacement [29]. The resonator is simplified as a clamped-clamped beam as shown in Figure 6.

After the bending deformation, the beam’s tension *F_l_* could be calculated as:

(4) $$F_{l}=ES\frac{\partial L}{L_{0}}$$

where *E* is the silicon’s Young’s modulus, *S* is the cross-section area of the beam, while *L*_0_ and *L* are the initial length of the beam and its length after deformation, respectively. Since the deformation is slight enough when compared with its length, the deformed beam can be approximated as a straight beam. As a result, the beam’s length after the bending deformation could be expressed as:

(5) $$L=\sqrt{L_{0}^{2}+4y^{2}}=L_{0}\sqrt{1+{\left(\frac{2y}{L_{0}}\right)}^{2}}=L_{0}[1+2{\left(\frac{y}{L_{0}}\right)}^{2}+o(y^{2})]$$

where *y* is the displacement of the beam’s midpoint. Its mechanical tension *F_y_* could be calculated as:

(6) $$F_{y}=2F_{l}\sin\alpha \approx 2F_{l}\tan\alpha =\frac{8ES}{L_{0}^{3}}y^{3}+o(y^{3})$$

In this resonator, sensitive masses are mounted at *L*_0_/4 from the anchor point, and the bending movement’s turning center is *L*_0_/8 from the anchor point. Therefore, the bending arm *L_y_* = 3*L*_0_/8 and the bending angle *ϕ_y_* due to the deformation is:

(7) $$\phi_{y}\approx \tan\phi_{y}=\frac{y/2}{L_{0}/8}=\frac{4y}{L_{0}}$$

Finally, we can get the expression of the additional mechanical moment:

(8) $$M_{y}=F_{y}L_{y}=\frac{3ESL_{0}}{64}\phi^{3}+o(\phi^{3})$$

As a result, the mechanical nonlinear elastic coefficients of the bending oscillation due to its structural deformation can be expresses as:

(9) $$\{\begin{array}{c} k_{m1}=0\\ k_{m2}=0\\ k_{m3}=\frac{3ESL_{0}}{64} \end{array}$$

#### 3.1.2. Electrostatic Nonlinear Elastic Coefficients

**Figure 7:**
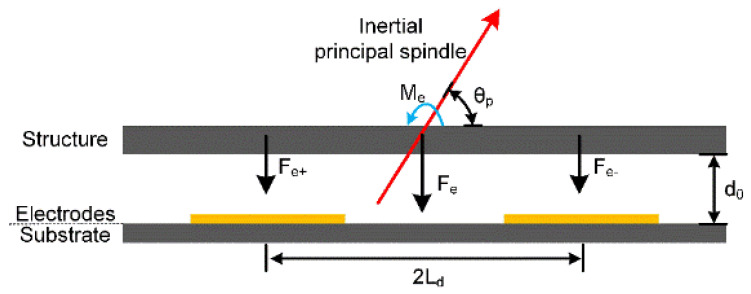
The schematic diagram of the resonator’s excitation principle.

The electrostatic nonlinearity in a capacitive resonator is mainly derived from high-order nonlinear electrostatic forces generated by large displacements [10]. This paper focuses on the resonator’s nonlinear electrostatic excitation forces and its schematic diagram is shown in Figure 7.

This device uses a differential excitation method so its electrostatic force could be calculated as:

(10) $$F_{e}=\frac{U_{+}^{2}}{2}\frac{\partial C}{\partial y}-\frac{U_{-}^{2}}{2}\frac{\partial C}{\partial y}=\frac{\varepsilon_{0}\varepsilon_{r}AU_{+}^{2}}{2{\left(d_{0}-y\right)}^{2}}-\frac{\varepsilon_{0}\varepsilon_{r}AU_{-}^{2}}{2{\left(d_{0}+y\right)}^{2}}$$

Here, *U_+_* = *U_d_ + U_a_cosωt*, *U_−_ = U_d_* − *U_a_cosωt*, *A* is the capacitance area, *y* is the oscillation displacement, *ε*_0_ is the vacuum dielectric constant, *ε_r_* is the free space’s permittivity, and *d*_0_ is the initial capacitance gap. Similarly, using the Taylor expansion method, Equation (10) could be rewritten as:

(11) $$F_{e}\approx \varepsilon_{0}\varepsilon_{r}A(2U_{d}U_{a}\cos\omega t)(\frac{1}{d_{0}^{2}}+\frac{3}{d_{0}^{4}}y^{2})+\varepsilon_{0}\varepsilon_{r}A(U_{d}^{2}+U_{{}_{a}}^{2}\cos^{2}\omega t)(\frac{2}{d_{0}^{3}}y+\frac{4}{d_{0}^{5}}y^{3})+o(y^{3})$$

Considering the resonator’s torsion motion, the relationship between its oscillation displacement and the torsion angle is *y* = *L_t_ϕ_t_*, where *L_t_* is the horizontal distance from the electrode’s center to the oblique beam. Therefore, when the number of excitation electrode couples is 2, the moment caused by the electrostatic force could be calculated as:

(12) $$M_{et}=-2F_{e}L_{t}\approx 2L_{t}^{2}[\varepsilon_{0}\varepsilon_{r}A(2U_{d}U_{a}\cos\omega t)(\frac{1}{d_{0}^{2}}+\frac{3}{d_{0}^{4}}\phi_{t}^{2})+\varepsilon_{0}\varepsilon_{r}A(U_{d}^{2}+U_{{}_{a}}^{2}\cos^{2}\omega t)(\frac{2}{d_{0}^{3}}\phi_{t}+\frac{4}{d_{0}^{5}}\phi_{t}^{3})+o(\phi_{t}^{3})]$$

Since *U_a_* << *U_d_* in the experiments, ignoring those little AC items, the resonator’s torsional modal electrostatic nonlinear elastic coefficients can be expressed as:

(13) $$\{\begin{array}{c} k_{e1}=-\frac{4\varepsilon \varepsilon_{0}A_{t}U_{d}^{2}L_{t}^{2}}{d_{0}^{3}}\\ k_{e2}=0\\ k_{e3}=-\frac{8\varepsilon \varepsilon_{0}A_{t}U_{d}^{2}L_{t}^{4}}{d_{0}^{5}} \end{array}$$

Considering its bending motion, there is *y* = *L_b_ϕ_b_cosθ_p_*, where *L_b_* is the horizontal distance from the electrode’s center to the oblique beam. Therefore, when the number of driving electrodes couples is 4, the moment caused by the electrostatic force could be calculated as:

(14) $$M_{eb}=-4F_{e}L_{b}\cos\theta_{p}\approx 4L_{b}^{2}\cos^{2}\theta_{p}[\varepsilon_{0}\varepsilon_{r}A(2U_{d}U_{a}\cos\omega t)(\frac{1}{d_{0}^{2}}+\frac{3}{d_{0}^{4}}\phi_{b}^{2})+\varepsilon_{0}\varepsilon_{r}A(U_{d}^{2}+U_{{}_{a}}^{2}\cos^{2}\omega t)(\frac{2}{d_{0}^{3}}\phi_{b}+\frac{4}{d_{0}^{5}}\phi_{b}^{3})+o(\phi_{b}^{3})]$$

Similarly, its bending modal electrostatic nonlinear elastic coefficients can be expressed as:

(15) $$\{\begin{array}{c} k_{e1}=-\frac{8\varepsilon_{0}\varepsilon_{r}A_{b}U_{d}^{2}L_{b}^{2}\cos^{2}\theta_{p}}{d_{0}^{3}}\\ k_{e2}=0\\ k_{e3}=-\frac{16\varepsilon_{0}\varepsilon_{r}A_{b}U_{d}^{2}L_{b}^{4}\cos^{4}\theta_{p}}{d_{0}^{5}} \end{array}$$

#### 3.1.3. The Asymmetric Torsion Modal Nonlinearity

The resonator’s asymmetric torsion mode is shown in Figure 3a. The displacement of its supporting beam is quite slight when compared with its length, so the mechanical nonlinearity due to its geometric deformation can be ignored. As a result, its nonlinear oscillation is mainly caused by the electrostatic nonlinearity. The inherent elastic coefficient *k_t0_* of the resonator’s asymmetric torsion mode can be obtained through the mechanical analysis [30]:

(16) $$k_{t0}=\frac{8GI_{t}}{L_{0}}\approx \frac{8G\beta w_{0}^{3}h_{0}}{L_{0}}$$

where *G* is silicon’s shear modulus, *I_t_*, *β*, *w*_0_, *h_0_,* and *L*_0_ is the oblique beam’s inertia polar moment, torsion coefficient, width, height, and length, respectively. The nonlinear elastic coefficients of the resonator’s asymmetric torsion mode could be expressed as:

(17) $$\{\begin{array}{c} k_{t1}=k_{e1}=-\frac{4\varepsilon_{0}\varepsilon_{r}A_{t}U_{d}^{2}L_{s}^{2}}{d_{0}^{3}}\\ k_{t3}=k_{e3}=-\frac{8\varepsilon_{0}\varepsilon_{r}A_{t}U_{d}^{2}L_{s}^{4}}{d_{0}^{5}} \end{array}$$

Based on its actual structure and experimental settings, we can get the parameter values needed for theoretical calculation Equation (2), as shown in Table 2.

**Figure 8:**
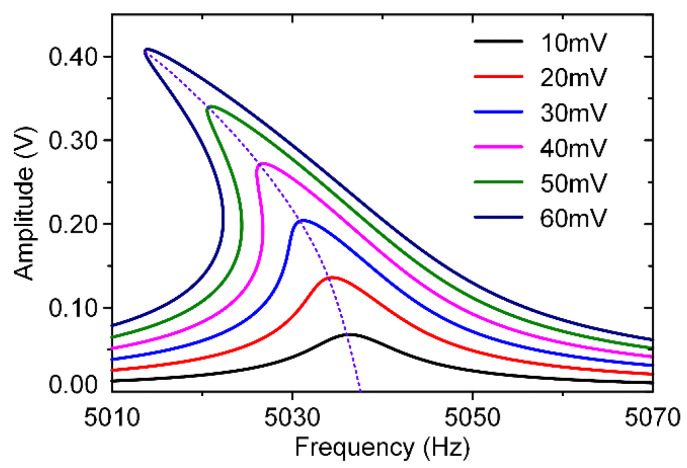
The theoretical analysis curves for the resonator’s asymmetric torsion mode.

Therefore, the resonator’s asymmetric torsion modal A-F curves with different AC voltages could be simulated as shown in Figure 8. It is obvious that the asymmetric torsion mode exhibits a typical stiffness softening effect.

#### 3.1.4. The Symmetric Bending Modal Nonlinearity

The resonator’s symmetric bending mode is shown in Figure 3b. The geometric deformation of the resonator’s supporting beam could not be ignored. Its nonlinear oscillation is caused by the combination of the mechanical nonlinearity and the electrostatic nonlinearity. As a result, the influence of these two nonlinearities on elastic coefficients must be considered together. The inherent elastic coefficient *k_b0_* can be obtained through the mechanical analysis [30]:

(18) $$k_{b0}=\frac{8EI_{w}}{L_{0}}\approx \frac{2w_{0}^{3}h_{0}E}{3L_{0}}$$

where *I_w_*, *w*_0_, *h*_0_, and *L*_0_ is the oblique beam’s main inertia moment, width, height, and length, respectively. Its nonlinear elastic coefficients could be expressed as:

(19) $$\{\begin{array}{c} k_{b1}=k_{e1}=-\frac{8\varepsilon_{0}\varepsilon_{r}A_{b}U_{d}^{2}L_{b}^{2}\cos^{2}\theta_{p}}{d_{0}^{3}}\\ k_{b3}=k_{m3}+k_{e3}=\frac{3ESL_{0}}{64}-\frac{16\varepsilon_{0}\varepsilon_{r}A_{b}U_{d}^{2}L_{b}^{4}\cos^{4}\theta_{p}}{d_{0}^{5}} \end{array}$$

Based on its actual structure and experimental settings, we can get the parameter values needed for theoretical calculation Equation (2), as shown in Table 3.

**Figure 9:**
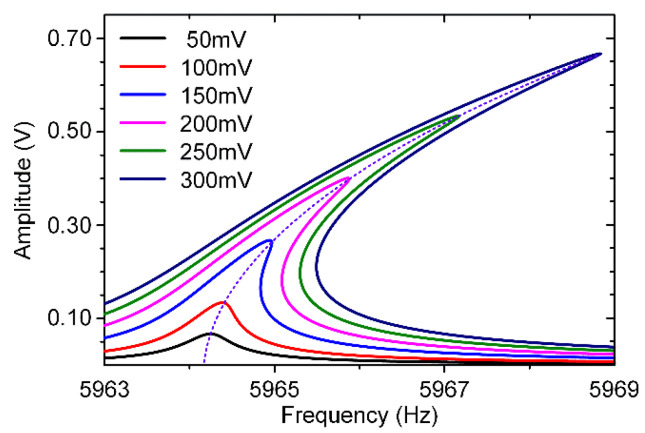
The theoretical analysis curves for the resonator’s symmetric bending mode.

Therefore, the resonator’s symmetric bending modal A–F curves with different AC voltages could be simulated as shown in Figure 9. It is obvious that the symmetric bending mode exhibits a typical stiffness hardening effect.

Obviously, through the special structural design, the resonator’s asymmetric torsion mode and symmetric bending mode have opposite nonlinearities, making it an ideal experimental device for analyzing the electrostatic modal coupling effect in the stiffness hardening region.

### 3.2. Electrostatic Modal Coupling Effect

**Figure 10:**
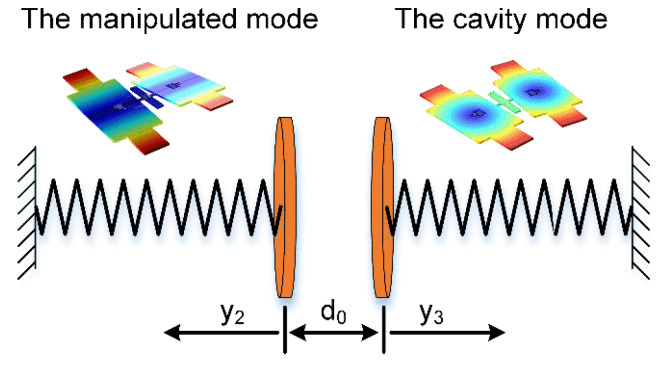
The schematic diagram of the electrostatic modal coupling effect in a capacitive micromechanical resonator. As for this resonator, its asymmetric torsion mode is set as the manipulated mode while the symmetric bending mode is the cavity mode.

It has been proved in previous researches that capacitors can achieve the dispersive electrostatic modal coupling effect [19]. The capacitive coupling could be described by the equivalent model in Figure 10. The essence of the electrostatic modal coupling effect is those shared electrodes with a certain bias voltage. One mode’s vibration will change the capacitive gap periodically, resulting in the change of electric potential energy. This produces a periodic change in other mechanical mode’s equivalent stiffness. In this case, these modes are no longer independent of each other, but coupled. 

In order to avoid the pull-in effect, the resonator’s vibrational angles are limited in a small range. The change of the capacitive area can be ignored. As a result, when two modes are excited together, the system’s kinetic and potential energy could be expressed as:

(20) $$\{\begin{array}{c} U=\frac{k_{2}\Phi_{2}^{2}}{2}+\frac{k_{3}\Phi_{3}^{2}}{2}-\frac{A\varepsilon_{0}\Delta V^{2}}{2(d_{0}+a\Phi_{2}+b\Phi_{3})}+\frac{3ESL_{0}}{256}\Phi_{3}^{4}\\ T=\frac{I_{2}{\dot{\Phi }}_{2}^{2}}{2}+\frac{I_{3}{\dot{\Phi }}_{3}^{2}}{2} \end{array}$$

where *k_j_*, *I_j_*, and Φ*_j_* (j = 2, 3) are the resonator’s inherent stiffness, inertia moment, and oscillational angle, respectively. ∆*V* is the bias voltage on shared electrodes, while *a* and *b* are linear motion coefficients. Using the Lagrange method, the coupled system’s dynamic equations could be expressed as:

(21) $$\{\begin{array}{c} I_{2}{\ddot{\Phi }}_{2}+k_{2}\Phi_{2}+\frac{A\varepsilon_{0}\varepsilon_{r}\Delta V^{2}}{2{\left(d_{0}+a\Phi_{2}+b\Phi_{3}\right)}^{2}}=0\\ I_{3}{\ddot{\Phi }}_{3}+k_{3}\Phi_{3}+\frac{A\varepsilon_{0}\varepsilon_{r}\Delta V^{2}}{2{\left(d_{0}+a\Phi_{2}+b\Phi_{3}\right)}^{2}}+\frac{3ESL_{0}}{64}\Phi_{3}^{3}=0 \end{array}$$

Introduce damping terms and expand nonlinear terms, Equation (21) can be rewritten as:

(22) $$\{\begin{array}{c} {\ddot{\phi }}_{2}+\gamma_{2}{\dot{\phi }}_{2}+\omega_{2}^{2}\phi_{2}+\alpha_{2}\phi_{3}+\beta_{2}{\left(\phi_{2}+\phi_{3}\right)}^{2}+\nu_{2}{\left(\phi_{2}+\phi_{3}\right)}^{3}=M_{2}\cos(\omega_{2}t)\\ {\ddot{\phi }}_{3}+\gamma_{3}{\dot{\phi }}_{3}+\omega_{3}^{2}\phi_{3}+\alpha_{3}\phi_{2}+\beta_{3}{\left(\phi_{2}+\phi_{3}\right)}^{2}+\nu_{3}{\left(\phi_{2}+\phi_{3}\right)}^{3}+\xi \phi_{3}^{3}=M_{3}\cos(\omega_{3}t) \end{array}$$

Based on the multi-scale method [24], the relationship of coupling modes can be calculated as:

(23) $$\begin{array}{l} m_{2}^{2}=\frac{\omega_{2}^{2}{\left|\phi_{2}\right|}^{2}}{\gamma_{2}^{2}}+{\left(\frac{1}{4}\Lambda_{2}{\left|\phi_{2}\right|}^{3}+\frac{1}{4}\Pi_{2}\left|\phi_{2}\right|{\left|\phi_{3}\right|}^{2}+\frac{2\omega_{2}\left|\phi_{2}\right|\sigma_{2}}{\gamma_{2}}\right)}^{2}\\ m_{3}^{2}=\frac{\gamma_{3}^{2}\omega_{3}^{2}{\left|\phi_{3}\right|}^{2}}{\gamma_{2}^{4}}+{\left(\frac{1}{4}\Lambda_{3}{\left|\phi_{3}\right|}^{3}+\frac{1}{4}\Pi_{3}{\left|\phi_{2}\right|}^{2}\left|\phi_{3}\right|+\frac{2\omega_{3}\left|\phi_{3}\right|\sigma_{3}}{\gamma_{2}}\right)}^{2} \end{array}$$

where,

(24) $$\begin{array}{l} \sigma_{2}=\frac{\omega_{d-2}-\omega_{2}}{\gamma_{2}},\sigma_{3}=\frac{\omega_{d-3}-\omega_{3}}{\gamma_{2}}\\ \Lambda_{2}=-\frac{2a^{3}b\beta_{2}\beta_{3}}{\left(4\omega_{2}^{2}-\omega_{3}^{2}\right)\gamma_{2}^{2}}-\frac{4a^{4}\beta_{2}^{2}}{3\omega_{2}^{2}\gamma_{2}^{2}}-\frac{2a^{3}b\beta_{2}\beta_{3}}{\omega_{3}^{2}\gamma_{2}^{2}}-\frac{3a^{3}\upsilon_{2}}{\gamma_{2}^{2}}\approx -\frac{3a^{3}\upsilon_{2}}{\gamma_{2}^{2}}\\ \Pi_{2}=-\frac{8a^{2}b^{2}\beta_{2}^{2}}{\left(\omega_{3}^{2}-4\omega_{2}^{2}\right)\gamma_{2}^{2}}-\frac{8ab^{3}\beta_{2}\beta_{3}}{\left(\omega_{2}^{2}-4\omega_{3}^{2}\right)\gamma_{2}^{2}}+\frac{2ab^{3}\beta_{2}\beta_{3}}{\omega_{3}^{2}\gamma_{2}^{2}}+\frac{2a^{2}b^{2}\beta_{2}^{2}}{\omega_{2}^{2}\gamma_{2}^{2}}-\frac{6ab^{2}\upsilon_{2}}{\gamma_{2}^{2}}\approx -\frac{6ab^{2}\upsilon_{2}}{\gamma_{2}^{2}}\\ \Lambda_{3}=-\frac{2ab^{3}\beta_{2}\beta_{3}}{\left(4\omega_{3}^{2}-\omega_{2}^{2}\right)\gamma_{2}^{2}}+\frac{4b^{4}\beta_{3}^{2}}{3\omega_{3}^{2}\gamma_{2}^{2}}+\frac{2ab^{3}\beta_{2}\beta_{3}}{\omega_{2}^{2}\gamma_{2}^{2}}-\frac{3b^{3}\upsilon_{3}}{\gamma_{2}^{2}}-\frac{3\xi_{3}}{\gamma_{2}^{2}}\approx -\frac{3b^{3}\upsilon_{3}}{\gamma_{2}^{2}}-\frac{3\xi_{3}}{\gamma_{2}^{2}}\\ \Pi_{3}=-\frac{8a^{2}b^{2}\beta_{3}^{2}}{\left(\omega_{2}^{2}-4\omega_{3}^{2}\right)\gamma_{2}^{2}}-\frac{8a^{3}b\beta_{2}\beta_{3}}{\left(\omega_{3}^{2}-4\omega_{2}^{2}\right)\gamma_{2}^{2}}+\frac{2a^{2}b^{2}\beta_{3}^{2}}{\omega_{3}^{2}\gamma_{2}^{2}}+\frac{2a^{3}b\beta_{2}\beta_{3}}{\omega_{2}^{2}\gamma_{2}^{2}}+-\frac{6a^{2}b\upsilon_{3}}{\gamma_{2}^{2}}\approx -\frac{6a^{2}b\upsilon_{3}}{\gamma_{2}^{2}}\\ m_{2}=\frac{M_{2}}{\gamma_{2}^{2}},m_{3}=\frac{M_{3}}{\gamma_{2}^{2}} \end{array}$$

Based on its actual structure and experimental settings, we can get the parameter values needed for theoretical calculation Equation (23), as shown in Table 4.

**Figure 11:**
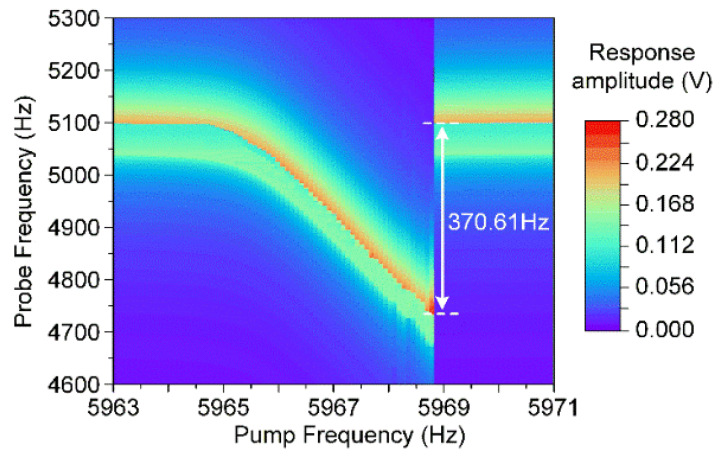
Simulation of the dispersive frequency shift of the resonator’s asymmetric torsion mode when simultaneously actuate its symmetric bending mode.

As a result, the electrostatic modal coupling effect could be simulated by numerically solving Equation (23) and the simulation result is displayed in Figure 11. Obviously, the manipulated mode’s fundamental frequency shifts 370.61 Hz under the influence of the cavity mode. It shows that the electrostatic modal coupling effect has a great potential to tune the frequency in a wide range.

## 4. Experiments and Discussion

Throughout the experiments, the device was placed in a temperature-controlled chamber and maintained at a constant temperature of 303.15 K. Before experimental tests, the resonator pre-operated for 2 h under the above constant temperature condition, and then data started to be collected. In this way, start-up errors and temperature drift errors can be greatly reduced.

### 4.1. Nonlinearity Experiments

**Figure 12:**
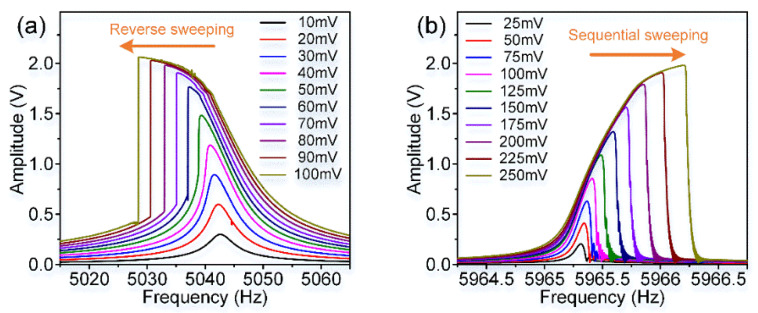
The amplitude-frequency response curves of the resonator’s different modes. (**a**) The asymmetric torsion mode’s A-F curves; (**b**) The symmetric bending mode’s A-F curves.

In these experiments, a stable 6.5 V DC voltage and different AC excitation signals produced by the lock-in amplifier are applied on these excitation electrodes as shown in Figure 4. The dynamic adjustment of electrostatic forces can be achieved by changing AC voltages. Figure 12 displays the amplitude-frequency(A-F) responses of the resonator’s asymmetric torsion mode and symmetric bending mode when separately driven by different AC voltages.

As shown in Figure 12, the resonator’s asymmetric torsion mode and symmetric bending mode exhibit different nonlinearities, which are consistent with theoretical simulation results. Obviously, as the increase of excitation voltages, the resonator’s nonlinearities become more serious. Its frequency drift is positively correlated with the voltage change. The asymmetric torsion mode’s electrostatic nonlinearity accounts for a greater proportion than its mechanical nonlinearity, showing a “stiffness softening” effect. On the contrary, the resonator’s symmetric bending mode exhibits a “stiffness hardening” effect, where its mechanical nonlinearity is stronger than electrostatic nonlinearity. The nonlinear characteristic of this specially designed resonator’s modes is completely different, which is of great significance for the identification of the resonator’s modal coupling responses.

### 4.2. Modal Coupling Experiments

**Figure 13:**
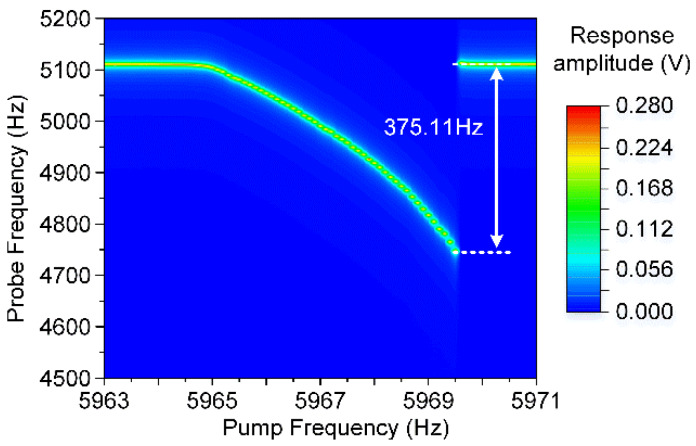
The dispersive modal coupling effect between the resonator’s asymmetric torsion mode and symmetric bending mode.

In the modal coupling experiments, this novel resonator’s asymmetric torsion mode and symmetric bending mode are simultaneously actuated by the probe and pump electrodes, respectively. The asymmetric torsion mode is excited in its stiffness softening region with an AC excitation voltage of 50 mV, while the symmetric bending mode is in its stiffness hardening condition with a pump signal of 250 mV. In this case, the resonator’s manipulated mode and cavity mode are excited in different nonlinear conditions. The manipulated mode’s responses at different pump frequencies are recorded, and a typical dispersive modal coupling effect is observed in Figure 13.

Obviously, with the increment of the pump frequencies, the asymmetric torsion mode’s fundamental frequency shifts to a low frequency region, which is consistent with the theoretical simulation result. The frequency shift of the manipulated mode is about 375.11 Hz, nearly 90 times its mechanical bandwidth 4.2 Hz. As a result, it has been proved from the experiments and analysis that the electrostatic modal coupling effect is a good method to tune the frequency difference between two coupled modes. Moreover, the frequency hopscotch of the manipulated mode also appears in the latter stage consistent with the condition when the cavity mode shows a mechanical nonlinearity.

### 4.3. Discussion

It is obvious that when the related parameters are the same, the results of theoretical simulation and experimental tests are consistent, which proves the correctness of theoretical models.

According to the electrostatic coupling theory, it is reasonable that the manipulated mode’s frequency will change with the cavity pump mode’s motion [31]. The cavity mode’s vibration will change the gap between the resonant structure and shared electrodes. At this time, since coupled modes share the same resonant structure and electrodes, their displacements are superposed. The cavity pump mode will generate an electrostatic negative stiffness in the manipulated mode, causing its frequency to shift [19]. As a result, the resonant frequency of the manipulated mode is modified by the cavity pump mode’s vibration. In this case, the electrostatic modal coupling effect could be used as a novel frequency tuning method in the most of capacitive micromechanical resonators.

Furthermore, it should be noted that the manipulated mode’s frequency hopscotch is quite different from the conventional electrostatic modal coupling frequency hopscotch. In the conventional electrostatic modal coupling, the electrostatic nonlinearity dominates in these coupled modes. When these electrostatic nonlinear modes couples, the manipulated mode’s frequency hopscotch appears in the initial region as shown in Figure 4d from Reference [19]. However, when it comes to the novel capacitive resonator used in this paper, its manipulated mode is electrostatic nonlinearity, while the cavity mode is a typical mechanical nonlinear mode. Since the cavity mode operates in a mechanical nonlinear state, the frequency hopscotch of the manipulated mode appears at the latter stage after a slowing decreasing region as shown in Figure 11. It is apparently found that the nonlinearity of the cavity mode plays a key role in the appearance of the frequency hopscotch.

This frequency shifting difference could also be explained theoretically. Based on Equation (23), it is easy to get the maximum frequency shift of the coupled modes *σ_jmax_*:

(25) $$\{\begin{array}{c} \sigma_{2\max}=-\frac{\gamma_{2}^{3}}{8\omega_{2}}\left[\frac{\Lambda_{2}m_{2}^{2}}{\omega_{2}^{2}}+\frac{\Pi_{2}m_{3}^{2}\gamma_{2}^{2}}{\omega_{3}^{2}\gamma_{3}^{2}}\right]\\ \sigma_{3\max}=-\frac{\gamma_{II}^{3}}{8\omega_{III}}\left[\frac{\Lambda_{3}m_{3}^{2}\gamma_{2}^{2}}{\omega_{3}^{2}\gamma_{3}^{2}}+\frac{\Pi_{3}m_{2}^{2}}{\omega_{2}^{2}}\right] \end{array}$$

It can be obtained that frequency shift directions of coupled modes are determined by *Λ*_2_, *Π*_2_ and *Λ*_3_, *Π*_3_. These key parameters are exactly the reflection of coupled modes’ nonlinear elastic coefficients as shown in Equation (24), which means that the frequency shift direction of each mode is dominated by its own nonlinearity. When the mode is dominated by the electrostatic nonlinearity, its frequency will shift downward, while in the mechanical nonlinear condition, it will shift upward.

As for the frequency hopscotch of the manipulated mode, it depends on the nonlinearity of the cavity mode. When the cavity mode is a mechanical nonlinear mode, its fundamental frequency will shift to a higher region. During the pumping process, the manipulated mode will encounter the resonant frequency of the cavity mode in the latter stage so that its frequency hopscotch appears in the latter stage. In this case, the manipulated mode’s frequency will slightly decrease with the increment of the pump signal at first. Then the frequency hopscotch appears and the manipulated mode’s frequency jumps back to its initial value in the latter stage as shown in Figure 11. However, when the cavity mode is an electrostatic nonlinear mode, its fundamental frequency will shift to a lower region so that the manipulated mode’s frequency hopscotch appears in the initial stage. In this condition, the manipulated mode’s frequency jumps to a lower frequency in the initial stage and then slowly rises back to its initial frequency as shown in Figure 4d from the Reference [19].

It is demonstrated that the frequency shifts of modes are closely related to the state of the manipulated mode and the cavity mode, which determined the shift direction and frequency hopscotch, respectively. This electrostatic modal coupling model is suitable for the most capacitive micromechanical resonators, especially when the coupled modes have different types of nonlinearities.

## 5. Conclusions

The modal coupling effect in a novel nonlinear micromechanical resonator is studied in this paper. To analyze the electrostatic modal coupling effect with different nonlinear coupled modes, a novel tuning fork resonator is specially designed and fabricated. In this capacitive micromechanical resonator, due to its unique structure size and excitation method, its main operating modes show the mechanical nonlinearity and the electrostatic nonlinearity, respectively.

It is worth noting that due to the existence of shared electrodes, the resonator’s intrinsic modes are no longer independent of each other, but coupled together. Therefore, the oscillation of any mode will modulate the other modes’ states. The displacement of the cavity pump mode will cause an electrostatic negative stiffness in the manipulated mode, causing its frequency shifting. Meanwhile, the shift direction is determined by the sign of the manipulated mode’s nonlinear coefficient. It has been demonstrated that the frequency shift range is normally much larger than its mechanical bandwidth, indicating that it could be used to tune the frequency in a wide range.

Moreover, this paper also compares the effect of different nonlinear cavity modes on the frequency shifting. It has been found that the nonlinearity of the cavity mode dominates the location of the manipulated mode’s frequency hopscotch. When the cavity mode is a mechanical nonlinear mode, during the pumping process, the manipulated mode will encounter its frequency hopscotch in the latter stage. On the contrary, when the cavity mode is an electrostatic nonlinear mode, the manipulated mode’s frequency hopscotch will appear in the initial stage.

The dispersive modal coupling effect is a product of the coherent phonon manipulation based on the electrostatic coupling, and it has great potential in enhancing the sensor performance. It has been proven that it is a good method to tune the frequency difference between two coupled modes in a wide range. Further, this electrostatic modal coupling model presented in this work is suitable for most capacitive micromechanical resonators.

## Figures and Tables

**Table 1:** Comparison between the simulation results and experimental results.

Mode	Simulation Results	Experimental Results	Frequency Difference
Asymmetric torsion mode	4916 Hz	5042.5 Hz	126.5 Hz
Symmetric bending mode	5809 Hz	5965.3 Hz	156.3 Hz

**Table 2:** The parameters used in simulations of the asymmetric torsion modal nonlinearity.

*I_t_* (kg·m^2^)	*Q_t_* (1)	*k_t_*_0_ (N·m/rad)	*k_t_*_1_ (N·m/rad)	*k_t_*_3_ (N·m/rad^3^)	*U_d_* (V)
9.32 × 10^−14^	7524	2.42 × 10^−4^	−1.06 × 10^−6^	−0.37	6.5

**Table 3:** The parameters used in simulations of the symmetric bending modal nonlinearity.

*I_b_* (kg·m^2^)	*Q_b_*	*k_b_*_0_ (N·m/rad)	*k_b_*_1_ (N·m/rad)	*k*_*b*3_ (N·m/rad^3^)	*U_d_* (V)
9.32 × 10^−14^	14561	3.66 × 10^−4^	1.02 × 10^−9^	1.90 × 10^−3^	6.5

**Table 4:** The parameters used in simulations of the modal coupling effect.

*γ*_2_	*γ*_3_	*Λ*_2_	*Λ*_3_	*Π*_2_	*Π*_3_	*U_d_* (V)	*U_a2_* (V)	*U_a3_* (V)
4.21	2.57	2.25 × 10^14^	−3.33 × 10^9^	2.96 × 10^11^	2.96 × 10^11^	6.5	0.05	0.25
